# The missing large impact craters on Ceres

**DOI:** 10.1038/ncomms12257

**Published:** 2016-07-26

**Authors:** S. Marchi, A. I. Ermakov, C. A. Raymond, R. R. Fu, D. P. O'Brien, M. T. Bland, E. Ammannito, M. C. De Sanctis, T. Bowling, P. Schenk, J. E. C. Scully, D. L. Buczkowski, D. A. Williams, H. Hiesinger, C. T. Russell

**Affiliations:** 1 Southwest Research Institute, Boulder, Colorado 80305, USA; 2 Massachusetts Institute of Technology, Cambridge, Massachusetts 02139, USA; 3 Jet Propulsion Laboratory, California Institute of Technology, Pasadena, California 91109, USA; 4 Lamont-Doherty Earth Observatory, Columbia University, New York, New York 10968, USA; 5 Planetary Science Institute, Tucson, Arizona 85719, USA; 6 USGS Astrogeology Science Center, Flagstaff, Arizona 86001, USA; 7 Department of Earth and Space Sciences, University of California, Los Angeles, California 90095, USA; 8 Istituto di Astrofisica e Planetologia Spaziali, INAF, Roma 00133, Italy; 9 Department of Geophysical Sciences, University of Chicago, Chicago, Illinois 60637, USA; 10 Lunar and Planetary Institute, Houston, Texas 77058, USA; 11 John Hopkins Applied Physics Laboratory, Laurel, Maryland 20723, USA; 12 School of Earth and Space Exploration, Arizona State University, Tempe, Arizona 85287, USA; 13 Institut für Planetologie, Westfälische Wilhelms-Universität, Münster 48149, Germany

## Abstract

Asteroids provide fundamental clues to the formation and evolution of planetesimals. Collisional models based on the depletion of the primordial main belt of asteroids predict 10–15 craters >400 km should have formed on Ceres, the largest object between Mars and Jupiter, over the last 4.55 Gyr. Likewise, an extrapolation from the asteroid Vesta would require at least 6–7 such basins. However, Ceres' surface appears devoid of impact craters >∼280 km. Here, we show a significant depletion of cerean craters down to 100–150 km in diameter. The overall scarcity of recognizable large craters is incompatible with collisional models, even in the case of a late implantation of Ceres in the main belt, a possibility raised by the presence of ammoniated phyllosilicates. Our results indicate that a significant population of large craters has been obliterated, implying that long-wavelength topography viscously relaxed or that Ceres experienced protracted widespread resurfacing.

## Abstract (web summary)

Studying craters on atmosphere-less bodies can unlock information about planetesimal histories. Here, Marchi *et al*. present results from the NASA Dawn mission to Ceres showing that craters >100–150 km in size are largely absent, and find that Ceres' internal evolution is responsible for their absence.

## Graphical abstract

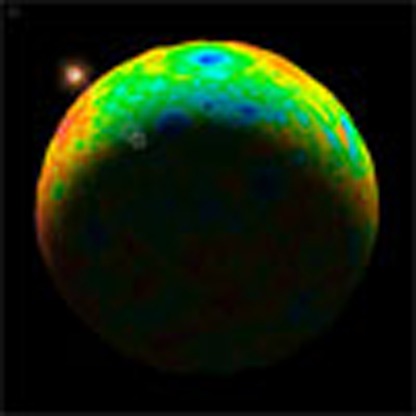

It is generally accepted that planets formed by the accretion of smaller building blocks that are referred to as planetesimals (for example, ref. 1). In the present solar system, main belt asteroids between Mars and Jupiter represent the leftovers of the initial population of planetesimals. As such, main belt asteroids have long been considered to retain important clues about the formation and collisional evolution of planetesimals^2^,^3^,^4^,^5^.

Ceres, with a mean diameter of 939 km, is by far the largest object in the main belt. Earlier observations concluded that Ceres is likely partially differentiated^6^, a prediction confirmed by gravity field measurements by the Dawn spacecraft^7^. As a result, it is believed that Ceres' accretion took place early in the solar system—possibly within a few Myr after condensation of the first solids—while radiogenic heating by^26^ Al decay was still extant. Therefore, it is likely that Ceres witnessed most of solar system evolution. Furthermore, because Ceres resides in a collisionally intense environment, impacts with nearby siblings are expected to be a primary evolutionary process for its outermost layers.

By virtue of its large cross section and ancient formation, Ceres offers a unique opportunity to study the earliest and most violent phase of collisional evolution by sampling craters whose sizes can largely exceed those on any other asteroid. As a comparison, the largest crater on 525 km asteroid Vesta—the second largest asteroid visited by a spacecraft—is ∼500 km across^8^. It is indeed commonly observed even among smaller asteroids that the largest recognizable crater approaches the size of the parent body^9^.

Ceres, however, lacks craters in excess of ∼280 km in diameter, and it is, as shown here, severely depleted in large craters down to 100–150 km in diameter. At the same time, large portions of Ceres' northern hemisphere are saturated with craters 60 km in diameter or smaller. These findings are utterly incompatible with current collisional models over a wide range of assumptions, including a late implantation of Ceres into the main belt, a possibility suggested by the discovery of widespread ammoniated phyllosilicates^10^,^11^,^12^. We conclude that a significant population of large cerean craters has been obliterated beyond recognition over geological time scales, likely the result of Ceres' peculiar composition and internal evolution. Finally, we present topographic evidence for the presence of one—possibly two— 800 km diameter depressions that may be relict impact basins from large collisions that took place early in Ceres history.

## Results

### Global crater distribution

**Figure 1:**
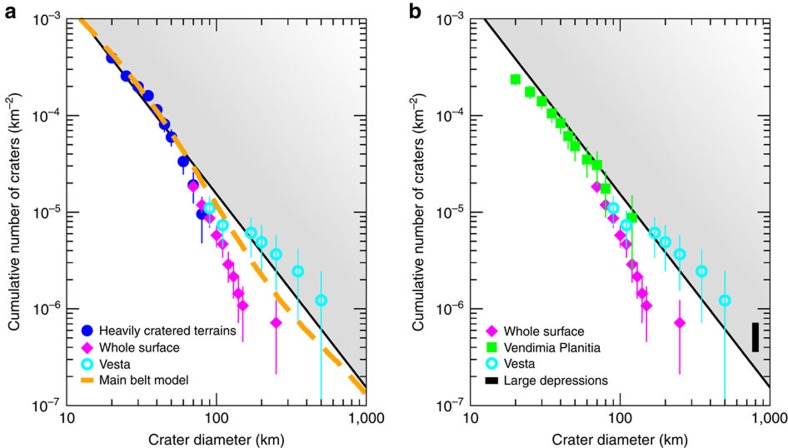
Ceres global large crater size-frequency distribution. (**a**) Cumulative crater counts on the whole surface for diameter >70 km (magenta). For a comparison, counts for the heavily cratered terrain (blue) are also reported: The drop-off at 70–80 km is due to the limited area of the counting region (for latitudes northern than 50° N, ∼15% of the surface). A model crater size-frequency distribution (dashed curve) is also reported (see Methods section). Note that the model curve has been vertically shifted to match the heavily cratered terrains at 20–60 km, thus it underestimates the number of large craters produced over 4.55 Gyr (see text). Black thin lines and shaded region mark empirical saturation, defined as 10% of the geometrical saturation^39^. (**b**) Crater cumulative counts for the northern part of Vendimia Planitia (green), as the southern part has been largely reset by the formation of Kerwan crater. The black vertical line indicates 1–2 basins of 800 km in diameter (see text). Vesta's large crater count^8^ is also indicated (cyan). Error bars correspond to Poisson statistics of the cumulative numbers.

Ceres' surface exhibits a plethora of impact craters, making cratering a primary geological and evolutionary process. The spatial density of craters >20 km, however, is very asymmetric and varies across the surface by a factor of ∼5, with the highest density found in the northern hemisphere (Fig. 1a). The latter have reached a level of crater spatial density, in the range 20–70 km, compatible with saturation. The global size-frequency distribution (SFD) for large craters (≥70 km) is shown in Fig. 1a. The spatial density of craters in the size range 70–80 km approaches that of the most heavily cratered terrain, found in the northern hemisphere. At crater sizes in the range ∼100–280 km, however, the global record of craters drops off, deviating significantly from predictions based on the current main belt asteroid SFD (Fig. 1a). The largest confirmed impact craters are Kerwan and Yalode, with approximate diameters of 280 and 270 km, respectively^13^.

Ceres' depleted large crater population is also at odds with that of Vesta, despite their similar collisional environment^14^. Vesta exhibits a significant number of larger craters (≥150 km; ref. 8), including two basins >400 km (Fig. 1a). Topographic evolution models based on an ice-rich shell indicate that cerean craters could be partially or completely viscously relaxed when compared with rocky bodies, such as Vesta^15^. However, Ceres' significant topography range (−7.3 km, +9.5 km) with respect to the 482 × 482 × 446 km reference ellipsoid^7^,^16^, locally high crater spatial densities (Fig. 1a) and high crater depth-to-diameter ratios^17^, argue against relaxation of an ice-rich shell as the primary cause for the lack of large craters.

### Ceres collisional evolution

**Figure 2:**
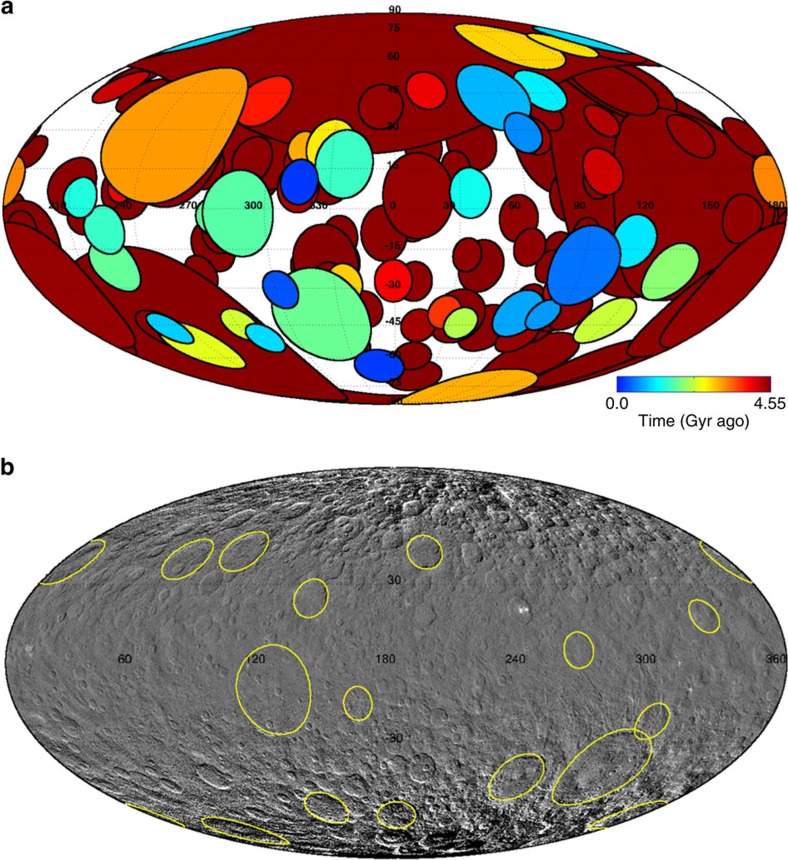
Simulated and observed large craters on Ceres. (**a**) Mollweide projection of all impact craters >100 km (∼170) expected to have formed since 4.55 Gyr ago. The picture shows a representative Monte Carlo simulation for our nominal model (see text). Colour code provides epoch of formation. The implementation of the Monte Carlo code follows a recent work^40^. The simulations track all the collisions with impactors >2 km, but here only the large ones are shown. While old craters are obliterated by subsequent cratering, empirical saturation shows that some 40 craters >100 km should be retained (Fig. 1). (**b**) Mollweide projection of a Ceres global mosaic showing observed 16 confirmed craters >100 km (yellow lines).

To quantitatively investigate the lack of large cerean craters, we ran a suite of Monte Carlo collisional models of Ceres assuming it had its current position in the main belt over the last 4.55 Gyr. The current estimated intrinsic collisional probability is *P*_i_=3.55 × 10^−18^ per year per km^2^, assumed to be constant through time (see Methods section). However, the total number of main belt asteroids, *N*_mb_(t), is expected to have been higher in the past^18^,^19^,^20^. As a result, the number of collisions per unit time per unit surface was also higher in the past, *N*_coll_(t)=(*P*_i_/4*π*) × *N*_mb_(t), where the factor 4*π* arises to express impacts per unit of target surface (see Methods section). Here, we apply a recent impact flux model calibrated at Vesta^21^ (see Methods section for details). Impactor sizes (*d*) were randomly drawn from the current main belt SFD, which is a reasonable approximation of the impactor size distribution over Ceres' history since the asteroid belt likely reached collisional equilibrium on a time scale <100 Myr (ref. 22). Finally, crater sizes (*D*) were computed using a crater scaling law^23^. For the crater size range of interest here (*D*∼100–800 km), the adopted scaling implies *D*∼10–13 × *d*, and we implemented a conservative factor of 10 for the nominal model. The results of the nominal model are shown in Fig. 2a. Furthermore, we investigate the effects of uncertainties in the crater scaling law by comparing it with alternate scaling laws^24^, and dedicated hydrocode impact simulations^25^. The latter results in the most conservative scaling for the final crater size, *D*=7.6 × *d*, which thus provides a lower bound for the number of craters at any given size (see Methods section). We ran ∼10^3^ simulations, and found that the model predicts the formation of about 90–180 craters >100 km, 40–70 >150 km and 9–14 >400 km. The range corresponds to *D/d*=7.6 and 10, respectively, as indicated above. Statistical uncertainties associated with our collisional model are within the indicated ranges. The total number of expected large craters indicates heavy reworking of the upper 10–15 km layers of Ceres, with important implications for the observed surface composition, geology and retention of crater morphology. In fact, the maximum number of detectable craters based on empirical saturation (Fig. 1a) is ∼40 and ∼20, respectively, for 100 and 150 km. At larger crater sizes, however, empirical saturation can be exceeded (as shown by Vesta—Fig. 1—and other asteroids^9^). Rescaling from Vesta by surface area, at least six to seven craters >400 km are expected.

The lack of well-defined large cerean basins is, therefore, remarkable. Assuming Poisson statistics, the likelihood of having no impact crater >400 km, if the average expectation is 6, is only 0.3%. In addition, the probability of observing 16 craters >100 km (Fig. 2b), if the observable average expectation is at least ∼40 (saturation), is vanishingly small.

### Searching for relict impact structures

**Figure 3:**
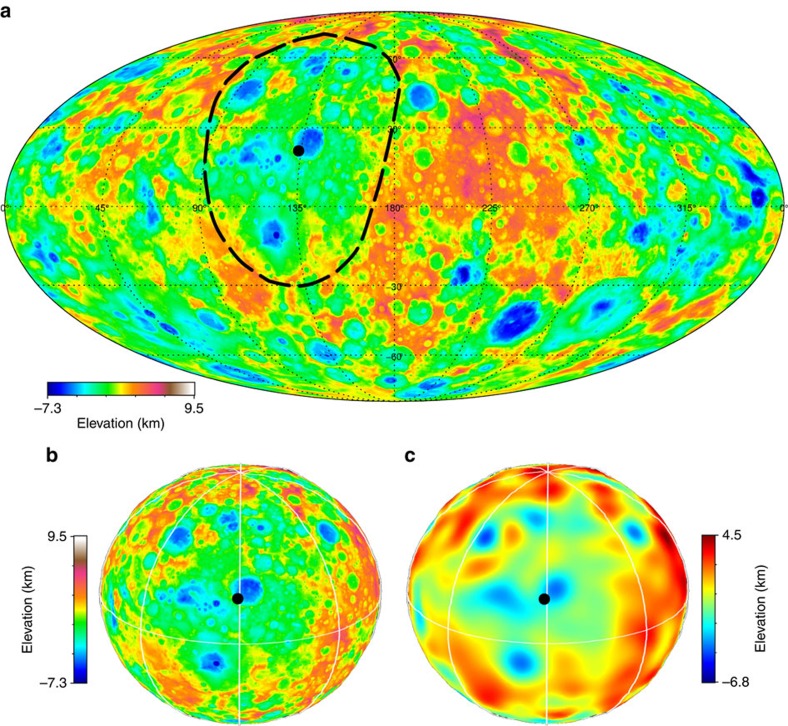
Ceres' Vendimia Planitia. (**a**) Mollweide projection of Ceres' topography. Dashed black line indicates a major depression or planitia. Black dots indicate the approximate centre of the depression. (**b**,**c**) 3D views of Vendimia Planitia in real and filtered topography, respectively. White lines indicate 45°-spaced meridians and the equator. Topography range is −7.3 to +9.5 km, while filtered topographic range is −6.8 to +4.5 km. The latter has been derived by applying a cosine taper to shape spherical harmonic coefficients between degree 15—corresponding to a scale of 200 km—and degree 30.

**Figure 4:**
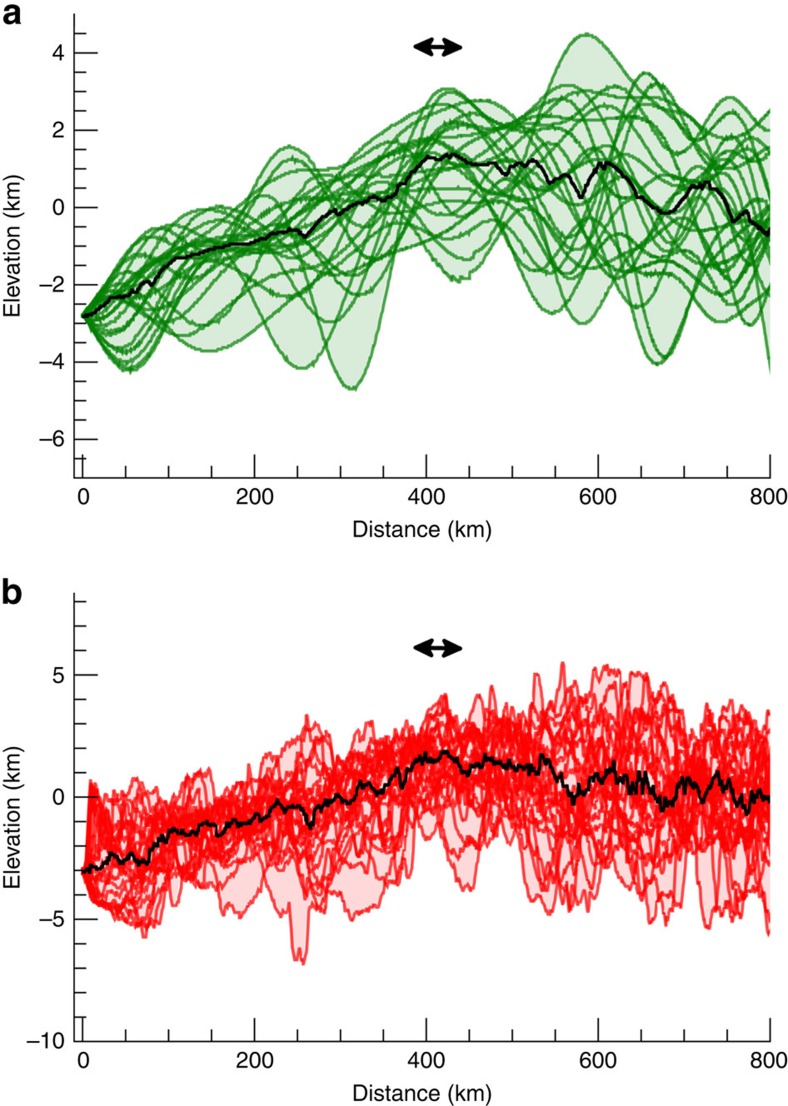
Topographic profiles for Vendimia Planitia. (**a**,**b**) Filtered and real topography, respectively. Black lines are the median of the profiles. Elevations are computed with respect to the 482 × 482 × 446 km ellipsoid^7^,^16^. Distance is computed along the surface of the best ellipsoid. Topographic profiles are for increments of 15° in azimuth, starting from the center of the feature (133°, 21° N) (see Fig. 3). Black arrows indicated the position of the rims.

These results provide the motivation for an in-depth search for large-scale impact structures. We used a global stereo-photogrammetry topography model, with a spatial resolution of 130 m px^−1^ acquired during the 1,475 km high altitude mapping orbit. Similar results are obtained with stereo-photoclinometry topography. Overall, most of the Ceres' surface is peppered by craters < ∼100 km, resulting in rugged topography, possibly hiding older, larger impact structures. Therefore, we use low-pass filtered topography to identify possible relict basins. A few quasi-circular and large-scale depressions, here referred to as *planitiae*, emerge. The most notable of these, Vendimia Planitia, is ∼800 km across (Fig. 3). Despite the fact that parts of the rim and floor are interrupted by superposed large craters, most notably by Kerwan and Dantu, the overall structure appears well defined. Radial profiles of filtered topography show a 3–4 km deep depression (Fig. 4a). A similar profile is also derived from the actual topography (Fig. 4b). Notably, the average topographic profiles show a distinctive conic-shaped trend, the presence of a raised rim and a gradual drop in elevation at increasing radial distances. These are all distinct markers for an impact structure. The overall shape of Vendimia Planitia's topographic profiles resembles that of the comparatively well-preserved Kerwan crater, if one takes into account minor post-formation evolution due to relaxation^17^ (see Methods section). Overall, this depression is the best candidate for a large crater recognizable on Ceres' surface (two other examples of putative basins are discussed in the Methods section).

It is noteworthy to mention that significant variations in spectral properties relative to Ceres' average spectrum are consistently found within Vendimia Planitia. For instance, a map of the absorption band at 3.1 μm, characteristic of ammoniated phyllosilicates^10^,^11^, shows a broad area of enhanced absorption (interpreted as enriched in ammoniated phyllosilicates) that correlates with the large depression (see Methods section). It is conceivable that impact excavation could have exposed materials with a different composition and/or triggered local alteration processes. The 3.1 μm absorption is prominent in the ejecta of the young Dantu crater, indicating that spectral differences are not just due to a thin surface layer, but are deep-seated. In addition, Ceres' Bouguer gravity (residual to the gravity of a two-layer structure with uniform density layers) and topography are generally anti-correlated^26^. Thus, areas of low topography—such as Vendimia Planitia, with an anomaly of 50 mGal—that are here interpreted as putative basins are underlain by relatively higher density material, indicating some degree of isostatic compensation. Craters superposed on this planitia are close to saturation, although somewhat below the heavily cratered terrains (Fig. 1b). This indicates that Vendimia Planitia is a very ancient feature, and if it is an impact basin, it is potentially responsible for the proposed Ceres' paleo-family^27^. Note that the presence of 1–2 800 km basins is in line with extrapolation from the Vesta's crater SFD (Fig. 1b).

## Discussion

The recent detection of widespread ammoniated phyllosilicates on Ceres has raised the possibility that Ceres could have been implanted in the main belt from the outer solar system^10^. Thus, this poses the question of whether a late implantation of Ceres into the main belt's collisionally intense environment could explain the observed lack of large craters. From a dynamical standpoint, the latest possible time for such implantation to take place is during the instability responsible for the so-called late heavy bombardment at 4.1–4.2 Gyr ago (refs 5, 28). Because the post-instability dynamical evolution of the main belt is well understood, we can constrain the cratering history since this phase reasonably well^29^.

Following the computations described above, and assuming no collisions before implantation, the computed number of craters since 4.1 Gyr ago to the present time is 24–43, 10–17 and 3–4, respectively for 100, 150 and 400 km diameters. While the presence of one (up to three) large-scale depressions discussed above could be compatible with a late implantation, the depletion in the 100–400 km size range is an overall very low probability event (<2%). Note that the above computation neglects cratering before implantation, thus it is a very conservative estimate. Furthermore, the cerean large crater SFD (*D*>100 km) does not match the main belt SFD (Fig. 1a). Thus, we can conclude that Ceres' cratering is not due to a late implantation in the main belt. Likewise, we can rule out that the observed cratering is due to primordial sublimation of a thick layer of ice, as this phase would have been concluded well before the late instability^30^.

The formation of a significant population of large craters, and their subsequent obliteration, is thus likely the result of Ceres' internal processes. For instance, viscous relaxation of long-wavelength topography may obscure very old impact basins, although short wavelength features such as rims are projected to survive over geological time scales, in analogy to the icy satellites (for example, ref. 31). It should also be noted that the presence of a thin low viscosity top layer, which could quickly remove rims, is ruled out by the locally high spatial density of km-sized craters. Alternatively, internal evolution models predict the possible presence of liquid water for hundreds of Myr after formation of first solids^32^,^33^, potentially resulting in protracted widespread cryovolcanism, whose last vestiges can perhaps be found in Ahuna Mons^34^, and the bright materials in Occator crater^12^. Widespread resurfacing could also provide an explanation for the pervasive presence of ammoniated phyllosilicates at the surface, as well as the lack of ancient crater rims. However, clear evidence for the presence of large-scale flow features is lacking at current image resolution. In addition to the aforementioned processes, rims could also be removed by the high spatial density of superposed smaller impacts.

Regardless of the specific mechanism(s) for crater rim removal, our result requires that large crater obliteration was active well after the late heavy bombardment. This conclusion reveals that Ceres' cratering record is inextricably linked to its peculiar composition and internal evolution.

## Methods

### Ceres ancient impact flux and crater scaling laws

**Figure 5:**
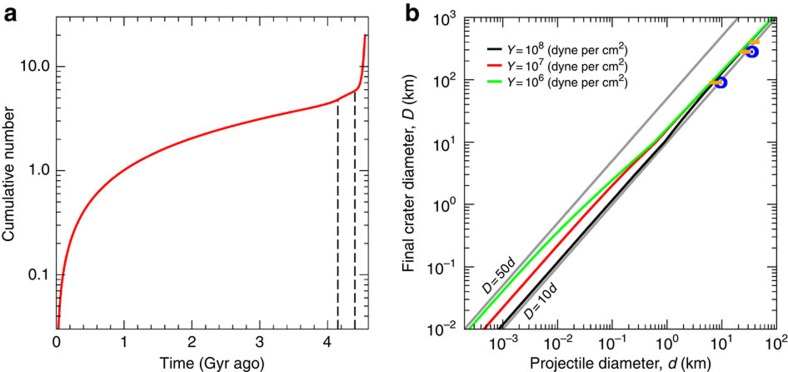
Model impact flux and crater scaling laws. (**a**) Cumulative number of impacts (normalized to 1 at 1 Gyr ago). Dashed lines indicate the approximate on-set of the late heavy bombardment (4.15 Gyr ago), and the end of the primordial depletion (4.4 Gyr ago), as described in the text. The shape of the impact flux curve is valid regardless of the impactor size, although it needs to be properly rescaled by the number of main belt asteroids larger than a given size to compute the actual number of collisions. (**b**) Solid curves correspond to the general crater scaling law of ref. 23 (their Table 1), corrected for the transient-to-final crater size (using a factor of 1.3 as discussed at http://keith.aa.washington.edu/craterdata/scaling/usermanual.html; complex crater sizes are further corrected using ref. 41). Other scaling law parameters are *ν*=0.4, *μ*=0.55, *K*_1_=0.93. Different colours (red, green and black) correspond to indicated values of target strength. Note that for *D* > ∼20 km, the effect of target strength becomes negligible, thus the actual value of strength does not affect our conclusions. As a reference, oblique grey lines indicate a constant scaling of *D*=10 × *d* and *D*=50 × *d*. Horizontal segments (orange) indicate results from the scaling laws summarized in ref. 24. They were computed using three crater diameter values as reference (92 km Occator; 280 km Kerwan and 400 km). The left-most extreme of each line corresponds to ref. 24 hard rock scaling, while the right-most value corresponds to porous scaling. Finally, we report the results of iSALE simulations^25^ of Ceres cratering calibrated to reproduce Occator and Kerwan crater's sizes (blues circles).

We estimated the collision probability of asteroids striking onto Ceres using the methods described in ref. 8. The resulting so-called intrinsic collision probability is *P*_i_=3.55 × 10^−18^ per year per km^2^, based on asteroids >50 km. Note that, following usual conventions, *P*_i_ is normalized to *r*^2^, where *r* is the radius of the target asteroid. Thus, the total number of impacts on an asteroid is *P*_i_ *r*^2^, and to calculate the number of impacts per unit area on the asteroid surface, this is divided by the asteroid surface area, *P*_i_ *r*^2^/(4*π r*^2^)=*P*_i_/(4*π*), which is the expression used in the main text. The time-dependent cumulative number of collisions, following ref. 21, is reported in Fig. 5a.

We implemented several crater scaling laws to test robustness of our conclusions. The details of this analysis are shown in Fig. 5b. In all cases, we used an average impact speed of 5.1 km s^−1^ (computed using the methods described in ref. 8), a target density of 2.1 g cm^−3^, and a projectile density of 2.6 g cm^−3^. Notice that the actual densities have a limited effect to the final crater sizes. For instance, a target density of 1.8 g cm^−3^ results in 5% larger craters, all other parameters being the same. Our adopted target density corresponds to Ceres' bulk value, thus it is likely an upper limit for the outermost layers. Higher target densities result in smaller crater sizes, thus it implies a conservative estimate for the final crater sizes. We also assumed a simple-to-complex crater transition of 10 km (ref. 7).

Among investigated crater scaling laws, iSALE simulations provided *D/d* ratios ranging from ∼9.4–10 to ∼7.6–8.2, respectively for Occator and Kerwan craters. We thus assumed the most conservative scaling of *D*=7.6 × *d*. The latter is used to compute a lower limit bound for the number of large craters predicted by our collisional models, as described in the main text.

### Topographic profiles for Kerwan and other large depressions

**Figure 6:**
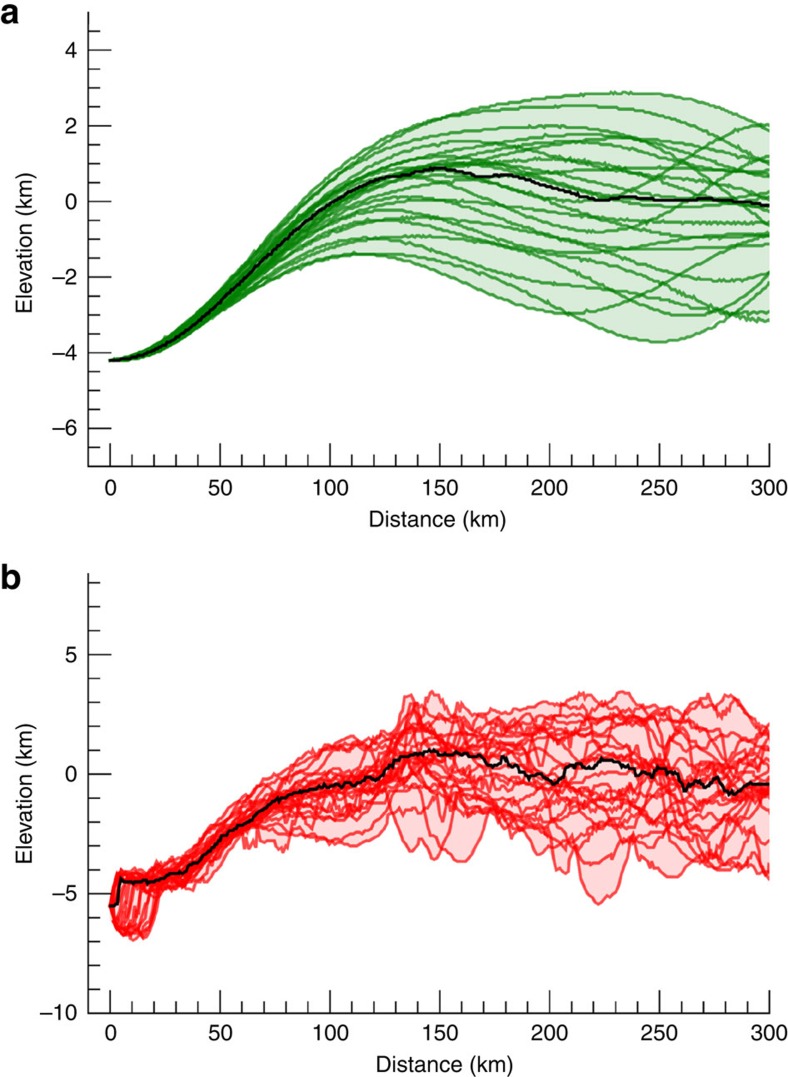
Kerwan crater's topographic profiles. (**a**) Filtered topography, (**b**) real topography. Black lines are the median of the profiles. Elevations are computed with respect to the 482 × 482 × 446 km ellipsoid^7^,^16^. Distance is computed along the surface of the best ellipsoid. Topographic profiles are for increments of 15° in azimuth starting from the centre of the feature.

Figure 6 reports the topographic profiles of Kerwan crater, the largest confirmed impact structure on Ceres. Note, the conic-shape profiles indicating that some degree of relaxation has taken place. These trends are qualitatively similar to those of Vendemia Planitia (Fig. 4), although the latter is certainly more relaxed due to an older formation age.

**Figure 7:**
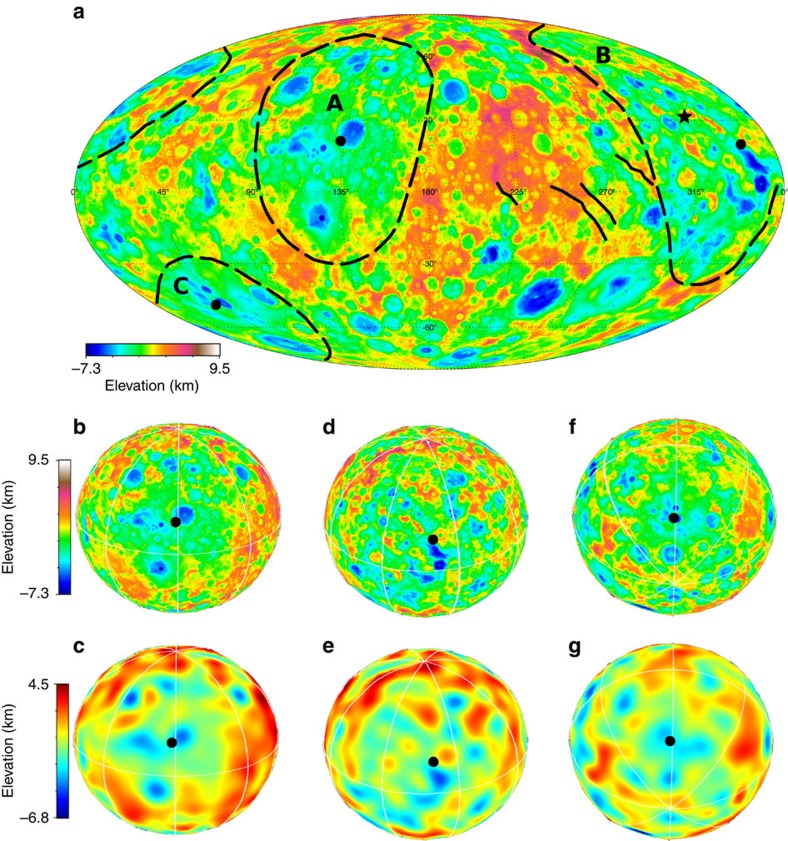
Planitia B and C. (**a**) Mollweide projection of Ceres' topography. (**b**,**c**) Real and filtered topography views centred at Vendimia Planitia, planitia B (**d**,**e**) and planitia C (**f**,**g**). Dashed black lines indicate the three major depressions or planitiae; while black dots indicate their approximate centres. (**b**–**g**) White lines indicate 45°-spaced meridians and the equator. Topography range is −7.3 to +9.5 km, while filtered topographic range is −6.8 to +4.5 km. The latter has been derived by applying a cosine taper to shape spherical harmonic coefficients between degree 15—corresponding to a scale of 200 km—and degree 30.

**Figure 8:**
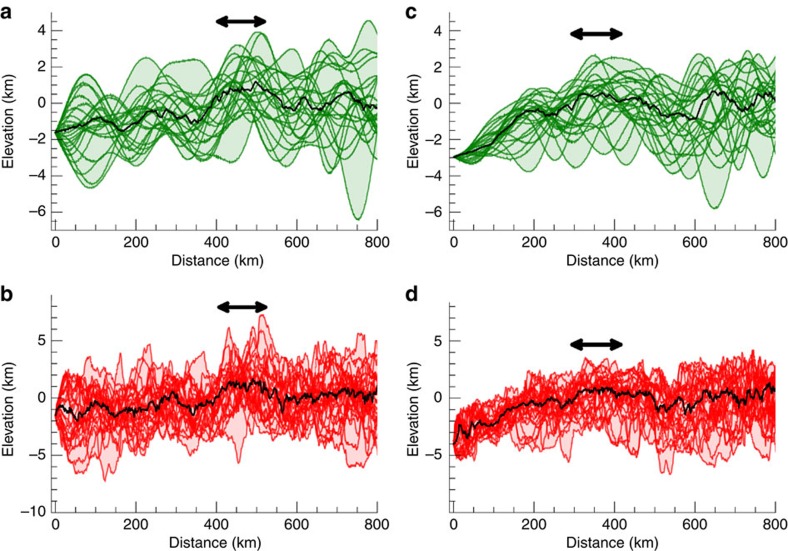
Planitia B and C topographic profiles. (**a**,**b**) Filtered and real topography for planitia B; (**c**,**d**) Filtered and real topography for planitia C. Black lines are the median of the profiles. Elevations are computed with respect to the 482 × 482 × 446 km ellipsoid^7^,^16^. Distance is computed along the surface of the best ellipsoid. Topographic profiles are for increments of 15° in azimuth starting from the centre of the feature. Black arrows indicated the position of the rims.

Additional large-scale depressions, indicated as Planitia B and C, are shown in Fig. 7. Planitia B is a 800 km depression delineated in the northern high latitudes by a remarkable arcuate ridge that extends for >1,000 km. Most of the southern rim is not visible, possibly due to the superposed cratering (for example, Yalode crater) or enhanced viscous relaxation at low latitude, with the exception of a short high relief segment whose relationship to the northern ridge is unclear. Topographic profiles across planitia B are noisier than those of Vendimia Planitia, although an average depression of 2–3 km with respect to the high relief ridge is observed (Fig. 8). In addition, the region immediately south of the putative centre of planitia B is the deepest depression on Ceres. A third, 500 km diameter feature, is also shown (planitia C). As before, topographic profiles show a consistent increase in elevation with increasing radial distance from the centre and a shallow raised rim (Fig. 8).

### Linear features and spectra variability

**Figure 9:**
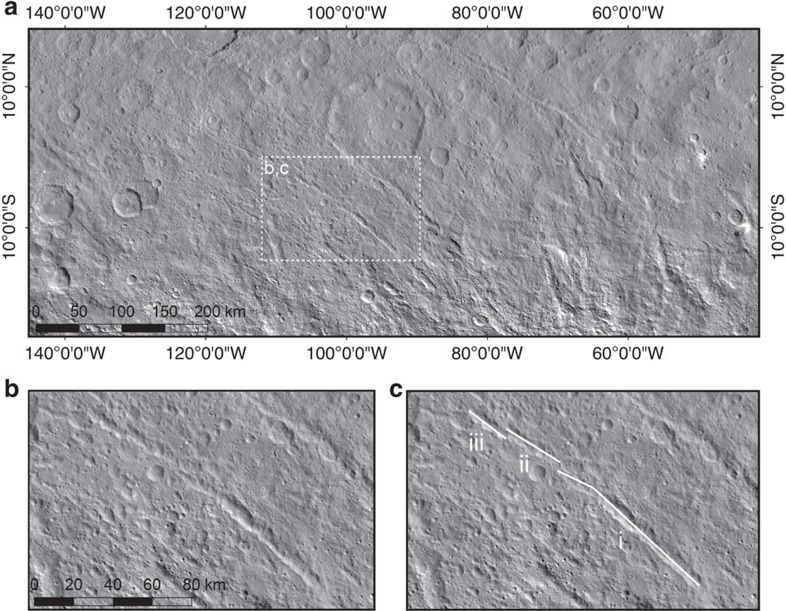
Pit chains south-west of planitia B. (**a**) Overview image of the set of pit chains whose pole plots in planitia B. (**b**,**c**) The blank (**b**) and annotated (**c**) inset images show part of the pit chain in the inset box in detail. The surface expression of the pit chain takes the form of merged pits in segment i while individual pits are still observed in segment ii and iii. The segments i, ii and iii are staggered/offset from one another, forming an en echelon pattern that is characteristic of fractures.

Notably, to the south-west of planitia B, there are clearly visible chains of pits (Fig. 7a; refs 16, 35). The pits are roughly circular depressions, however, unlike impact craters, they tend to lack clearly defined rims and partially coalesce into one another (Fig. 9). This observation, along with the observations that the pit chains form en echelon patterns, indicates that the pit chains formed as the surface expression of subsurface fractures. The pit chains range in width from ∼1.5 to ∼11 km, and range in length from ∼50 to ∼380 km. The average spacing of the pit chains is ∼84 km and the deepest pit is ∼1.5 km deep. These pit chains are cross-cut by ejecta and scour marks from Yalode, Urvara and Occator craters, which indicates that the corresponding subsurface fractures are older than the craters. We derive best-fit planes for these fractures using the orientation of the overlying pit chains and likely dip angles. For this, we used dip angles of ∼60–80°, based on the work of refs 36, 37, although further work is required to better constrain the geometry of the fractures. These planes return pole positions that are within planitia B (Fig. 7). This suggests that this depression could be an ancient impact structure, and that its formation may have fractured the surface of Ceres, which may be analogous to the formation of the Divalia Fossae and Saturnalia Fossae as a result of the Rheasivia and Veneneia impacts, respectively, on Vesta^38^; although in the latter cases, the dip angles were almost perpendicular to the surface.

**Figure 10:**
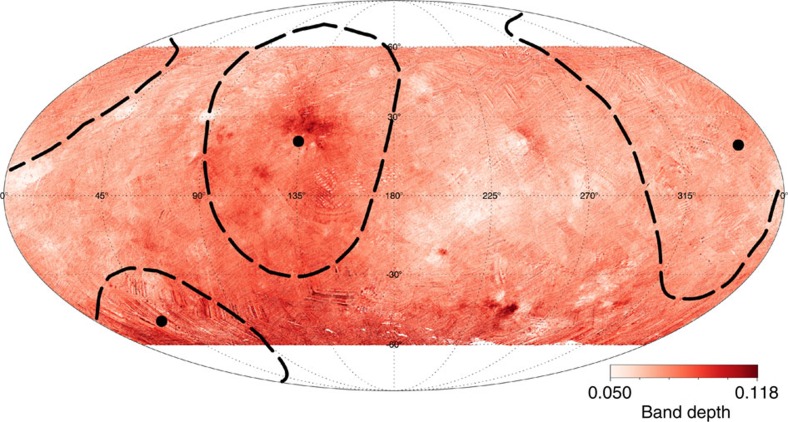
Global map of the ammoniated phyllosilicates absorption band. The colour shows the strength of the ammoniated phyllosilicates absorption band at 3.1 micron; dark colours indicate deeper band. Dashed lines indicate the boundaries of the three planitiae (see Fig. 7). The map is a Mollweide projection in the latitude range 60° S–60° N.

The 3.1 μm band also shows a broad, weak variation that correlates with planitia B, although less pronounced than for Vendimia Planitia (Fig. 10). The strength of the absorption band has been computed as illustrated in ref. 11. The input spectra have been filtered for illumination conditions and level of reflectance: only the spectra with incidence and emission angles <70°, phase angle between 15° and 60° and average I/F brightness >0.004 have been considered. To maximize the coverage, the map has been generated merging data sets with 3.5, 1.1 and 0.4 km px^−1^ spatial resolution (selecting at each location the best available resolution). However, other spectral maps do not show clear trends. An explanation could be that this is an older impact feature, consistent with the more subdued topography.

Finally, planitia B shows a clear correlation with a 50–100 mGal positive gravity anomaly and planitia C shows the strongest correlation to a >150 mgal positive gravity anomaly^26^, which is consistent with isostasy compensation of these features.

### Data availability

Crater counts and outputs of collisional simulations are available on request from the corresponding author.

## Additional information

**How to cite this article:** Marchi, S. *et al*. The missing large impact craters on Ceres. *Nat. Commun.* 7:12257 doi: 10.1038/ncomms12257 (2016).
